# A preventive integrated eHealth approach for individuals with a low socioeconomic position: protocol for a realist evaluation

**DOI:** 10.1186/s12889-024-20113-8

**Published:** 2024-10-03

**Authors:** Adriana M.C. Israel, Frank J. van Lenthe, Mariëlle A. Beenackers

**Affiliations:** Department of Public Health, Erasmus University Medical Center, P.O. Box 2040, Rotterdam, 3000 CA the Netherlands

**Keywords:** EHealth, Low socioeconomic position, Health behavior, Realist evaluation

## Abstract

**Background:**

Adoption of standalone eHealth tools is low among persons in lower socioeconomic groups. The preventive integrated eHealth approach combines blended care with an active and personal approach to facilitate access to local care, tailored to the needs of the participant. We describe the four step preventive integrated eHealth approach for individuals with a low socioeconomic position and the realist evaluation protocol of the intervention and implementation. The realist evaluation centers around the question, ‘what works for whom in what circumstances and why’.

**Methods:**

The study population will consist of adult individuals with a low socioeconomic position, who participate in the preventive integrated eHealth approach in one of the participating locations in the Netherlands. The four-step intervention consists of: (1) a proactive invitation of participants by care professionals, (2) the use of an eHealth tool that produces a personalized health report, (3) a personal consultation with a care professional to discuss the personalized health report and set a goal to work on, and (4) active referral to local social and health care. An initial program theory theorized from literature and stakeholder involvement is presented. Qualitative and quantitative data collection and analysis with participants (survey at zero, three and twelve months and focus groups at six months) and professionals (interviews at three months) will inform the realist evaluation and serves to test and refine the initial program theory.

**Discussion:**

Our mixed-methods realist evaluation on the effect and implementation of a personal and active blended care approach will elucidate what elements trigger the mechanisms and responses of how individuals with a low socioeconomic position experience the preventive integrated eHealth approach. This will inform the way a preventative health check incorporating eHealth can be used to its full potential for low socioeconomic positioned groups to help close the digital divide and contribute to reduce health disparities.

## Background

 Notable and well-documented health disparities exist across the socioeconomic gradient [1–3]. Unhealthy behaviors, abetted by unfavorable living circumstances, contribute to increased risks of non-communicable diseases in people with a lower socioeconomic position (SEP) [1, 2, 4, 5]. Adopting a healthier lifestyle, by means of increasing physical activity and healthy nutrition, reducing chronic stress, limiting alcohol intake, and quitting smoking, may help to reduce health disparities [6].

EHealth interventions for behavior change are becoming increasingly popular. These interventions offer potentially affordable, accessible, and personalized solutions for self-management [7, 8]. EHealth can empower patients and stimulate patient-centered care and engagement [9]. However, it requires a level of self-efficacy of the individual to engage in and play an active role in their own care process [10]. For lower SEP groups, a variety of adverse living conditions may impede on their ability to do so. Thus, it cannot be assumed that one size fits all [11–13].

Despite their effectiveness in promoting healthy behaviors in the general population or groups with a higher SEP, eHealth interventions in general have been less successful in populations with a lower SEP [7, 8, 14]. Among lower SEP groups are people with a low education, a low income or living in disadvantaged neighborhoods [15, 16]. The reach and adherence to such interventions is generally lower among persons with low as compared to a high SEP, and attrition rates are higher. This contributes to the reduced effectiveness observed for this group [7, 8, 17]. These observations could very well be explained by adverse socioeconomic conditions this group faces [17].

There exists a digital divide between groups with a lower and higher SEP. This divide extends to the use of eHealth [18, 19]. Factors like poverty or financial strain may limit access to necessary digital devices or stable internet [20]. Lower income and lower education are associated with lower digital skills levels which can complicate access to and the use of eHealth. Lower health literacy is also associated with these factors, making it more difficult to assess and interpret health information for individual use [19, 21, 22].

In addition, adverse socioeconomic conditions and limited resources can impede on the ability of an individual to adopt healthy lifestyles through the existence of chronic stress [11, 23, 24]. In such conditions, chronic stress can affect cognitive capacity and executive control, redirecting mental resources from long-term planning to immediate problem-solving. This can make it more difficult for individuals to change their health behaviors long term [11, 25, 26]. The existence of chronic stress and its consequences may come on top of less health consciousness and more fatalistic beliefs among lower SEP groups. Evidence suggests that this group experiences diminished self-control over health behaviors and their positive outcomes [26, 27].

Tailoring eHealth interventions specifically for individuals with low SEP seems to yield promising results [28]. Recent research on barriers and facilitators experienced by low SEP groups on adopting eHealth and lifestyle interventions provide insights into their specific needs [29–32]. If the aforementioned difficulties of digital and health literacy, diverse living conditions, and access are adequately addressed, increased use and adherence to lifestyle interventions using eHealth seems feasible for individuals with low SEP [17]. Especially user-centered eHealth that is attuned to the sociocultural and -economic conditions of the user is needed, with ways to tailor and personalize content to their individual needs [29, 30]. This includes content but also appropriate and easy to use language, supported by visual aids [30, 31].

Another key element that emerges from the literature, is the role of support of a care professional. While care professionals are regarded as trusted and credible sources for advice on behavior change, they are also trusted to advise on technically suitable and safe eHealth applications by vulnerable groups [30, 31, 33]. Reaching individuals with low SEP has been regarded as difficult. Yet active recruiting strategies with personal contact by care professionals through existing (social) networks have proven to be successful [30]. In addition, especially a blended care approach, combining eHealth components with face-to-face human interaction, is a great facilitator. A blended care approach stimulates engagement by the user through coaching and personal guidance and is a way to offer technical support when needed [31, 34, 35].

To address the difficulties lower SEP groups may experience, Dutch care professionals introduced the preventive integrated eHealth (PIE) approach to facilitate actions toward a healthier life. This four-step blended care approach is customized for individuals with a lower SEP. It integrates both online and offline elements for a complementary effect. The PIE approach consists of four steps: (1) proactive invitation of participants, (2) use of an eHealth tool that produces a personalized health report, (3) a personal consultation with a care professional, and (4) an active referral to local social and health care. Each step provides key elements that should be able to mitigate factors that inhibit a successful participation in the PIE-approach. These elements are: (1) facilitating access to the eHealth tool, (2) offering technical support and gaining insights from a personalized health report on the participant’s health behaviors, (3) coaching by a trusted care professional that takes into account the sociocultural and –economic living conditions, and (4) guidance to available local care. For participants, the last step of the PIE-approach may serve as a stepping stone to other interventions that tackle issues that affect a person’s health and health behaviors. However, this depends on the participant’s actions that follow after participation.

A comprehensive understanding of how complex health interventions like the PIE-approach elicit behavior change, requires research methodology which shifts from controlled settings to real-life practice. This focus on real-life practice is needed to incorporate the context in which participants engage with the intervention. The realist evaluation is such a methodology that goes beyond measuring outcomes and success or failure; it delves into the question of ‘What works for whom, in what circumstances, in what respects and how?’ [36]. This enables a deeper understanding of the underlying mechanisms, shedding light on what circumstances drive individuals with lower SEP to engage with eHealth interventions and how they are best supported with lifestyle change. Even though recent research has begun to determine barriers and facilitators experienced by low SEP groups, it remains unclear what specific components contribute to the effectiveness of interventions incorporating eHealth use for health behaviors. Realist evaluation allows for a theory-driven test of hypotheses to uncover what elements of an intervention elicit a behavior change response among subgroups in different contexts.

## The aims and objectives of the PIE-study

The aim of the present study is to understand how different elements of the PIE-approach create an impact for individuals with low SEP on their health and health behaviors. The objectives to reach this aim are:

I. To measure the impact of the PIE-approach on self-assessed overall health and well-being, health behaviors, underlying determinants and the intention to change health behaviors of individuals with low SEP;
II. To describe the perception of change of individuals with low SEP after participation in the PIE-approach and the acceptability and relevance of the PIE-approach for them;
III. To describe the contextual elements and underlying mechanisms linked to the outcomes of the PIE-approach;
IV. To validate the program theory from the perspective of individuals with low SEP and the care professionals who carry out the PIE-approach.

We will answer the research questions outlined in Table 1. to complete these objectives.

**Table 1:** Specific research questions to obtain objectives

Research questions
1. How were the different steps of the PIE-approach carried out for each location?
2. Who took part in the PIE-approach?
3. What different strategies for active and personal invitation and recruitment were used and how did they stimulate participation?
4. What effect did the use of an eHealth tool elicit among the participants and why?
5. What effect did the personal consultation with a care professional elicit among the participants and why?
6. What actions did the participants take after the PIE-approach and why?
7. What kind of barriers and facilitators did the participants and care professionals experience for each step of the PIE-approach and what mechanisms were triggered?
8. What was the outcome of the PIE-approach on self-assessed overall health and well-being, health behaviors, underlying determinants and the intention to change?

## Methods

### Realist evaluation

**Fig. 1:**
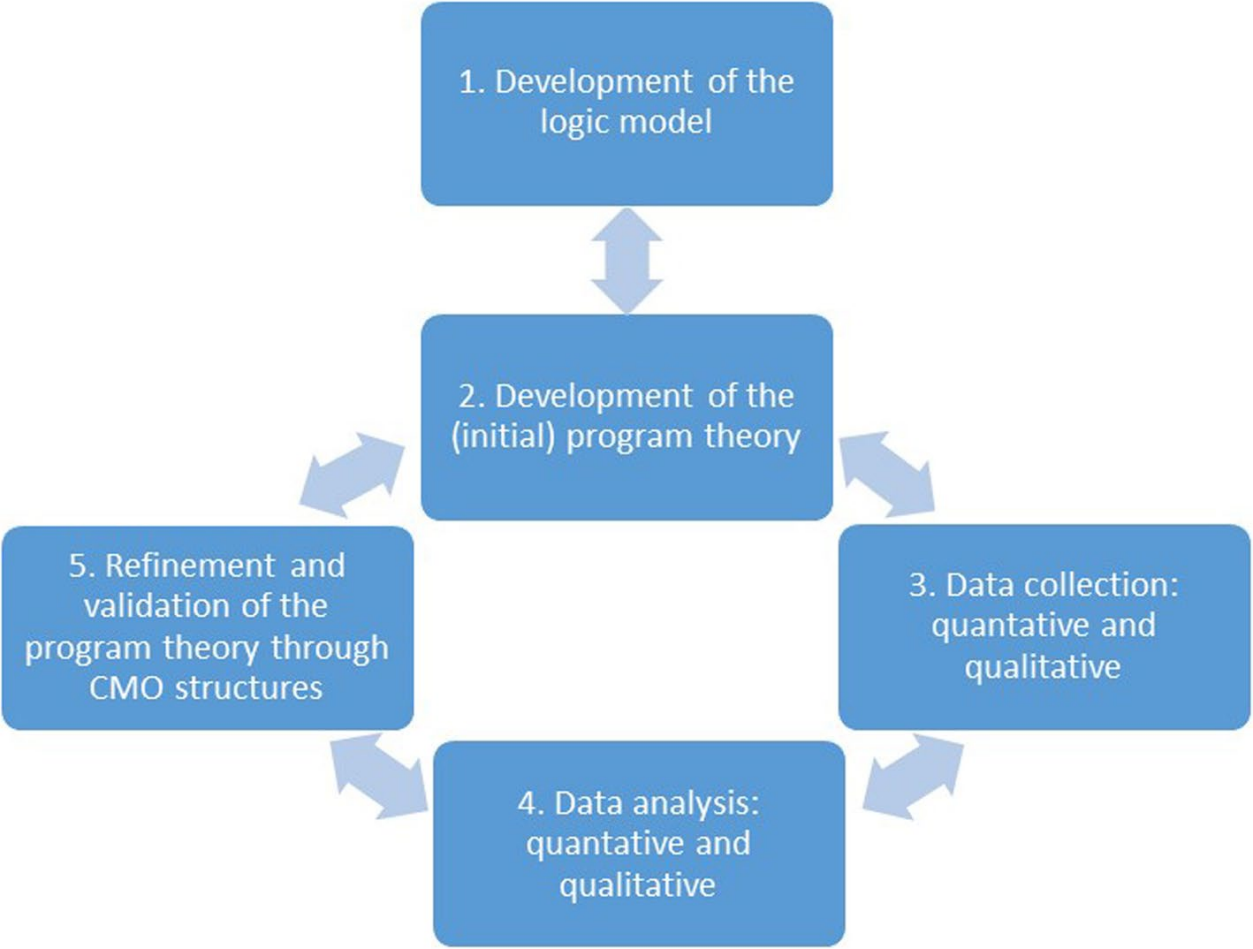
Five phases of realist evaluation. Based on processes described by Pawson & Tilley (1997) [36] and Salter & Kothari (2014) [37]. CMO: context-mechanism-outcome

This study performs a realist evaluation with a mixed methods design. The realist evaluation is a theory-driven method and chosen as it is suitable to evaluate complex interventions consisting of multiple elements [37]. It explicitly addresses the role context plays within implementation of a new intervention. The realist evaluation aims to validate and refine program theories on ‘what works for whom, in what circumstances, in what respects and how’ by testing initial program theories through context-mechanism-outcome configurations that are generated through data collection. The different phases of the realist evaluation are shown in Fig. 1. By means of combining quantitative methods, such as participant surveys, with qualitative methods, including focus groups with participants and semi-structured interviews with care professionals, it will be possible to describe the impact of the intervention on the participants and in what ways they engaged with the different elements. First the intervention is described in detail, after which the different phases of the process are explained.

### The PIE approach

**Fig. 2:**
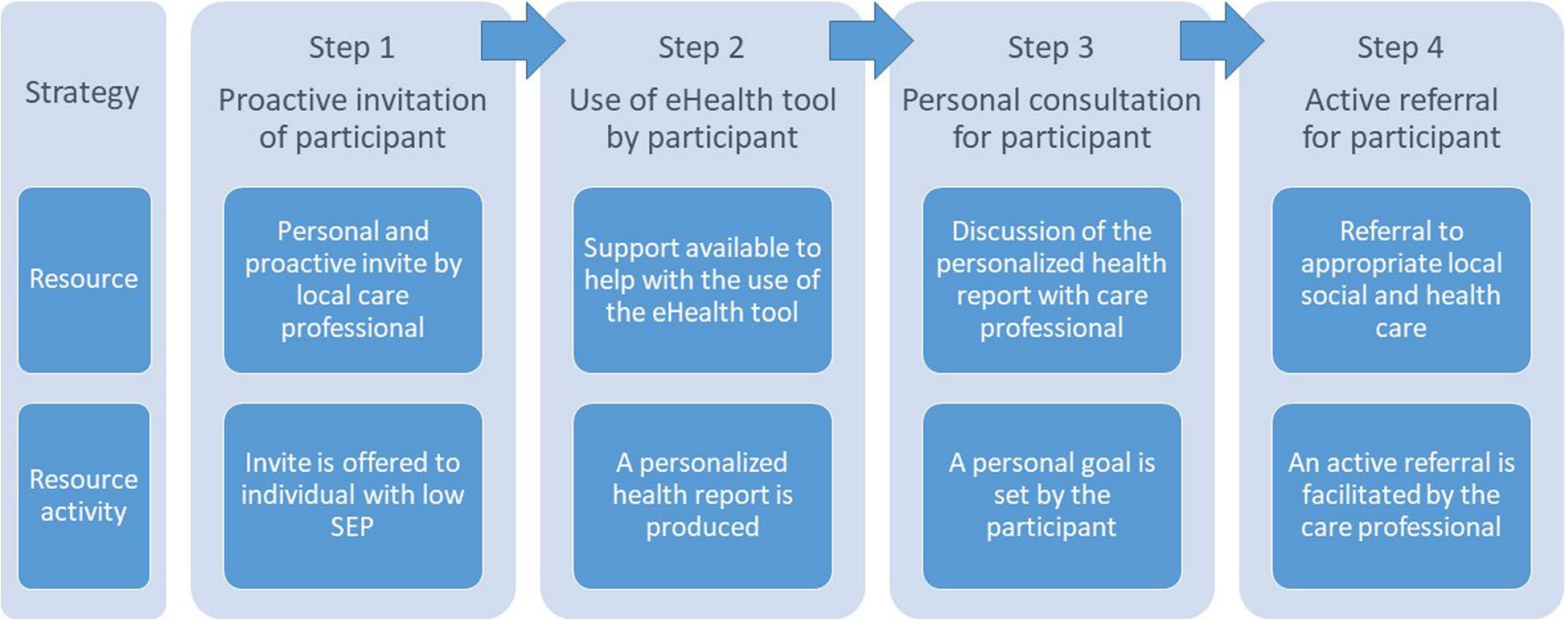
Figure 2: Four steps of the preventive integrated eHealth approach. SEP: socioeconomic position

The PIE-approach consists of the following four core elements: (1) proactive invitation of individuals with a low SEP, (2) the use of an eHealth tool that provides a personalized health report, (3) a personal consultation with a care professional and (4) an active referral to local social and health care (Fig. 2).

As step 1, care professionals that adopt and implement the PIE-approach will proactively invite participants of the target population to participate in the PIE-approach in a way that fits their local care practice. For example, a general practitioner can invite their patients from known deprived neighborhoods to come in for the PIE-approach as a general health check or a municipality can organize events within deprived neighborhoods where the PIE-approach is offered to its residents by care professionals. This proactive invitation of the target population ensures participation of persons who otherwise could be less inclined to participate in a preventive health check.

In step 2, an eHealth tool is used in order to support participants’ evaluation of their own health situation. The eHealth tool provides insight into a person’s health and health behaviors, and consists of an online questionnaire and a personalized health report. Either the care professional (or students under their supervision) or the researchers will assist the participant with making an account and filling out the online questionnaire. The use of an eHealth tool can help increase awareness and motivation among participants to set a personal goal that addresses issues that affect their health or health behaviors. The choice between two eHealth tools is up to the local stakeholders.

For step 3, the eHealth tool will be an integrated part of blended care as it is combined with an in person consultation with a care professional where they discuss the personalized health report. The participant and care professional will set a goal to work on for the participant, taking into account their resources, skills and living conditions, while acknowledgeing underlying causes of ill-health, such as financial strain or chronic stress. The care professional will provide advice on possibilities for local support resulting in step 4.

As step 4, the final step includes an active referral by the care professional of the participant to the local array of social or healthcare interventions and providers. An active referral means that, where appropriate and according to the participant’s wishes, the care professional will contact the organization to which they refer the participant to facilitate access. These interventions can include interventions related to the physical environment, behaviors and skills, social participation, prevention and care, social environment, financial circumstances or a combination of these.

### EHealth tools

The PIE-approach uses an eHealth tool, with the choice between the Personal Health Check (PHC), developed by Netherlands Institute for Prevention and EHealth Development (NIPED) or My Positive Health (MPH), developed by the Institution of Positive Health (IPH).

The PHC is a scientifically founded eHealth instrument, based on validated guidelines for personal health risk management, developed by NIPED in close collaboration with several Dutch healthcare and patient stakeholders [38]. The PHC aims to increase awareness and motivation among participants in order to take action for a healthier lifestyle. It provides insight into a person’s health and health behaviors and consists of an online questionnaire (~ 100 multiple choice questions), supplementary (home)tests and a personalized health report. The PHC was made more accessible for people with limited digital and information skills in collaboration with Pharos, the Dutch Centre of Expertise on Health Disparities.

The MPH is a digital communication tool developed by IPH [39]. Based on the research done by Huber to form a new definition of health, this tool is a digital questionnaire (44 rating scale questions) that results in an individual’s personal score on six different domains (bodily functions, mental functions & perceptions, spiritual dimension, quality of life, social & societal participation, daily functioning) in a spider web chart [39, 40]. It has been developed by care professionals to be used in practice as a conversation starter about personal views on self-perceived health and well-being.

### Phase 1 Development of the logic model

The first phase consists of constructing a logic model of the resources, program activities and outputs. The logic model (Table 2) was based on the initial experience with the PIE approach within a pilot project that was set up by care professionals and implemented in 2019 in the city of Amersfoort, the Netherlands. These resources and program activities were developed by care professionals based on their shared experiences and expert knowledge. The pilot project served as the foundation to be able to expand to multiple locations within this new research project.

**Table 2:** Logic model of the preventive integrated eHealth approach

Inputs	Processes	Outputs	Impact short term	Sustained impact/ long term outcomes
**Resources** • Multidisciplinary teams of care professionals in the field (GP, lifestyle coaches, social workers, pharmacists, municipality staff, exercise coaches, volunteers) • Training for use of eHealth tool by IPH/Niped • Intake by Pharos for implementation of the PIE-approach • Local networks that have access to persons with lower SEP • Funding • The use of a social map of local care initiatives/activities **Intervention** • The use of the Personal Health Check eHealth tool • The use of My Positive Health eHealth tool (and training for the use of positive health concept)	**Potential activities by staff** *Preparation phase* • Intake with Pharos for implementation questions • Training by IPH or NIPED *Intervention phase* 1) Inviting participants 2) Promoting/ facilitating use of the eHealth tools 3) Having personal consultations to explain results 4) Actively referring participants to local social and health care **Features offered in the intervention for participants** 1) Personal targeted invitation 2) Use of My Positive Health or Personal Health Check which produces a personalized health report 3) Goal setting on determinants of health during personal consultation 4) Active referral to local social and health care activities	**User uptake of intervention** • Participants get insight into their health status through use of eHealth tool • Participants set a personal goal to improve their determinants of health • Participants get useful and personalized advice from care professionals on how to accomplish their set goal in local social and health care • Participants receive an active referral from care professionals to local social and health care • Participants take action to complete set goal	**Outcome for participants exposed to the intervention** • Persons with lower SEP experience more self-efficacy; become more motivated and more health-conscious • Persons with lower SEP take action to improve their determinants of health • Persons with lower SEP know where to go locally if they have questions or want to take action to improve their determinants of health **Outcome for professionals active in the intervention** • Care professionals have consultations with persons with lower SEP • Care professionals have a good understanding of the local social map	**Outcome for community of exposure to the intervention** • Persons with lower SEP in lower SES neighborhoods know where to go if they want to work on their determinants of health • Persons with lower SEP have increased awareness of their health status and determinants and increased sense of well-being • Improved health outcomes for persons with lower SEP that participated in the PIE approach • Care professionals continue to have personal consultations with persons with lower SEP about their determinants of health • Care professionals know how to locally reach persons with lower SEP to offer help on improving their health determinants • Care professionals can guide persons with lower SEP to the right local facilities
Legend: GP: general practitioner; IPH: Institute for Positive Health, owner of My Positive Health eHealth tool; NIPED: Netherlands Institution for Prevention and EHealth Development, owner of Personal Health Check eHealth tool; SEP: socioeconomic position; MPH: My Positive Health eHealth tool; PHC: Personal Health Check eHealth tool; Pharos: a national knowledge institution on socioeconomic differences				

### Phase 2 Development of the initial program theory.

**Fig. 3:**
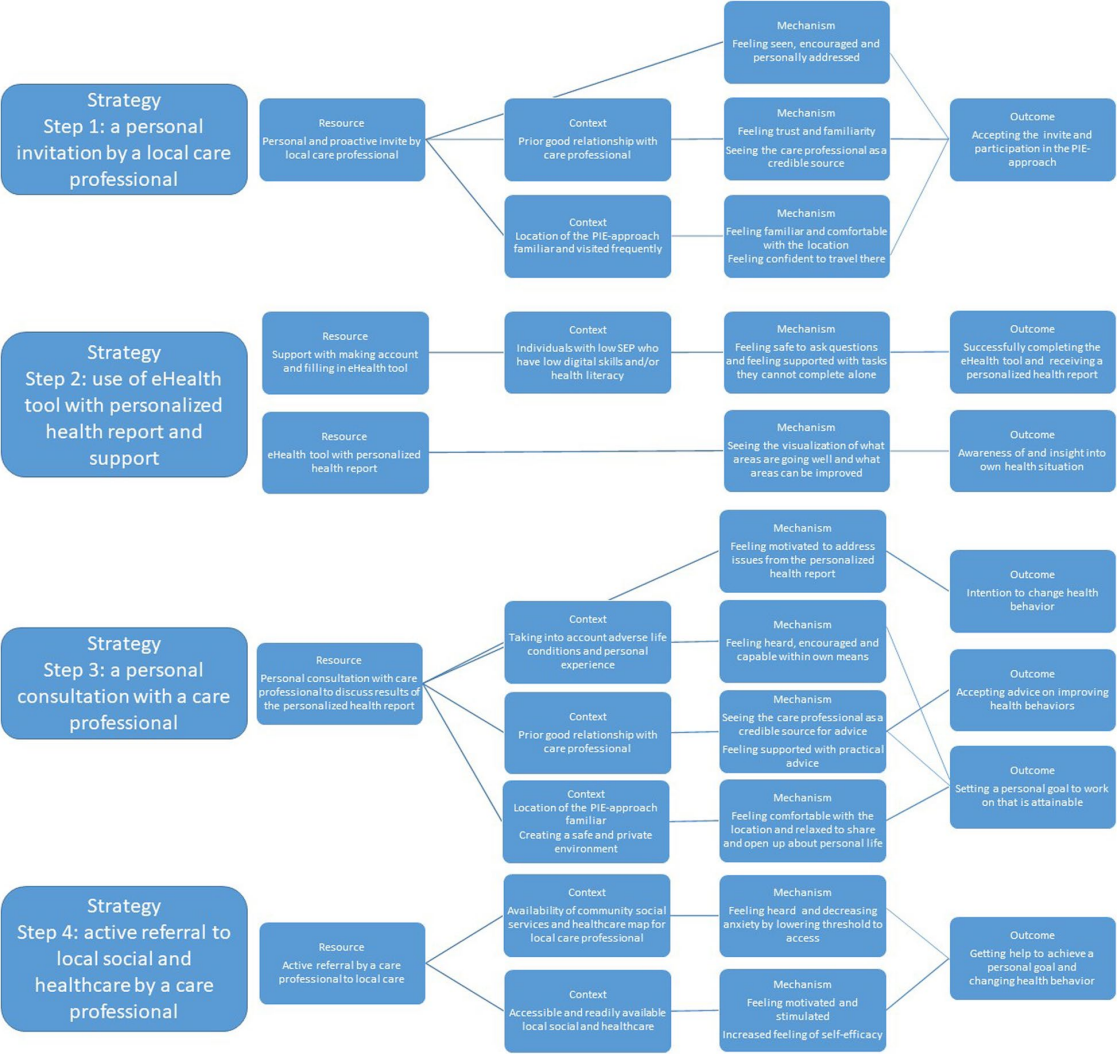
Initial program theory of the preventive integrated eHealth (PIE) approach

Our initial program theory (IPT) is based on the logic model presented in Table 2, two theories including the I-change model [41] and the Unified Theory of Acceptance and Use of Technology [42], and available literature examining specifically the attitudes, barriers, and facilitators among lower SEP groups in regards to use of eHealth and blended care [30, 44]. It should be noted that there was no program theory developed during the initial implementation of the PIE-approach in 2019. The logic model, theories and literature informed the IPT which is presented in Fig. 3. It is constructed according to the different steps and accompanying strategies of the PIE-approach. The IPT was subsequently discussed in an online meeting with different stakeholders such as care professionals and policy makers that have affinity and expertise on low SEP groups and eHealth. After consensus, this IPT, together with the logic model, was used as the starting point for the realist evaluation.

### Phase 3 Data collection

#### Study design and setting

The PIE study applies a quasi-experimental, non-randomized controlled trial design, with a follow-up period of 12 months within the realist evaluation. An intervention group of participants in the PIE-approach will be compared to a control group consisting of Dutch citizens who do not participate in the PIE-approach. The study will make use of natural field experiments (‘pilot locations’) in a maximum of 10 locations in the social or primary healthcare domain across the Netherlands. The pilot location will be similar in offering the four steps of the PIE-approach. However, they will differ in their local context, the way people are directed towards the eHealth tool, the specific support and guidance of participants and their aftercare, in line with local preferences and possibilities.

Recruitment of the pilot locations is facilitated through the network of VitaValley, an organization that focuses on innovation in preventative healthcare. Health disparities are becoming an increasing focus of Dutch government and municipality policies [44], which creates interest and funding to create experience with new tactics such as the PIE-approach. Social welfare and healthcare professionals who work with low SEP groups, and experience their daily struggles, have asked for national attention and sterner policies [45] to decrease health inequality. Together with the increased attention for preventive health strategies for non-communicable diseases, it is a topic that is of interest to many to ensure that scarce time, effort, and resources are directed towards effective care.

#### Study population

Adults (≥ 18 years) with low SEP living in the Netherlands are eligible for participation. Low SEP is defined by educational level, income or area of residence. For education, low SEP is based on the highest level of education obtained with a degree and defined as level 0–2 of the International Standard Classification of Education (ISCED) [46]. ISCED 0–2 includes people with elementary school or less, vocational practical education, lower secondary school, first classes of higher secondary education and lower vocational school [47]. For income, low SEP is defined as an income level not exceeding 120% of the social minimum income as noted by the Dutch government. For neighborhoods, low SEP is defined as neighborhoods that have a SES-WOA-score lower than 0,1. The SES-WOA-score is an aggregate socioeconomic status score based on the financial prosperity, educational level and work participation of households in this neighborhood. This score is calculated by the Statistics Netherlands for all of the Dutch municipalities [48].

Potential participants will be excluded if they are not of sufficient competence (i.e. do not have control over their own financial or care choices) or have mild cognitive impairment.

In addition to participants of the intervention, we invite care professionals and stakeholders who are part of the planning, implementation and organization of the PIE-approach to participate in the evaluation by means of semi-structured in-depths interviews.

#### Recruitment procedure PIE-study

All persons participating in the preventive integrated eHealth approach will be invited to participate in the PIE-study personally, either by the care professionals or the researchers. Potential participants receive an invitation letter with a link to a video explaining the study details and have the opportunity to ask the researchers questions about the study through email, telephone or in person, depending on way the intervention is offered locally. The control group will be recruited from neighborhoods with a similar socioeconomic profile according to their SES-WOA-score but which do not participate in offering the preventive integrated eHealth approach, through recruitment strategies specifically aimed at persons with low SEP. Eligible care professionals will be personally invited by the researcher to participate in an interview. Written or digital informed consent will be obtained from all participants prior to participating.

#### Data collection process

This mixed methods study makes use of quantitative methods in the form of digital or paper surveys for the participants at 0, 3 and 12 months after participants joined the PIE-approach. Qualitative methods include the use of four data sources: open questions in surveys and focus groups for participants, interviews with professionals, and ethnographic observation on location of pilots, including the scrutiny of policy and evaluation documents. The evaluation plan is presented in Table 3.

**Table 3:** Preventive integrated eHealth approach evaluation plan

Research question	Data collection	Data source
1. How were the different steps of the PIE-approach carried out for each location?	¬ Detailed description of resources used ¬ Data on reach, dose of intervention delivered and received	• Policy and evaluation documents • Ethnographic observation and logbook • Interviews with professionals
2. Who took part in the PIE-approach?	¬ Demographics: age, sex, migration background, educational level, occupational status, household income and composition, area of residence, digital literacy, health literacy, financial strain	• Participant questionnaire 0 months
3. What different strategies for active and personal invitation and recruitment were used and how do they stimulate participation?	¬ Detailed description of resources used ¬ Detailed description of responses to resources used and triggered mechanisms that lead to participation	• Policy and evaluation documents • Ethnographic observation and logbook • Interviews with professionals • Focus groups with participants
4. What effect did the use of an eHealth tool elicit among the participants and why?	¬ Detailed description of responses to resources used and triggered mechanisms regarding use of the eHealth tool	• Interviews with professionals • Focus groups with participants
5. What effect did the personal consultation with a care professional elicit among the participants and why?	¬ Detailed description of responses to resources used and triggered mechanisms regarding the personal consultation	• Interviews with professionals • Focus groups with participants
6. What actions did the participants take after the PIE-approach and why?	¬ Data on goals set and taken actions after the PIE-approach ¬ Detailed description of responses to resources used and triggered mechanisms that lead to goals set and taken actions	• Participant questionnaire 3, 12 months • Policy and evaluation documents • Interviews with professionals • Focus groups with participants
7. What kind of barriers and facilitators did participants and professionals experience for each step of the PIE-approach and what mechanisms were triggered?	¬ Data on appreciation of the steps of the PIE-approach ¬ Detailed description of responses to resources used and triggered mechanisms regarding positive and negative aspects	• Participant questionnaire 3, 12 months • Interviews with professionals • Focus groups with participants
8. What was the outcome of the PIE-approach on self-assessed overall health and well-being, health behaviors, underlying determinants and the intention to change?	¬ 17 items Positive Health measuring scale ¬ General self-assessed health, fruit and vegetable intake, weekly physical activity, duration of sleep, alcohol consumption, smoking habits, financial strain, stress inducing life events and perceived stress, intention to change health behavior, self-reported BMI	• Participant questionnaire 0, 3 and 12 months • Control group questionnaire 0, 3 and 12 months

#### Quantitative data collection

Participants will fill in a paper or digital survey at three moments (see Table 3). At T = 1 (0 months) the survey will include sociodemographic features, health and digital literacy, perceived stress including financial stress, self-assessed health and general well-being, and health related behaviors such as physical activity, vegetable and fruit intake, alcohol use, smoking and sleep. At T = 2 (3 months) and T = 3 (12 months) questions relating to stress, self-assessed health, and health behaviors will be repeated, with additional questions related to the process evaluation indicating participants’ experience with and appreciation of each element of the PIE-approach. The control group receive the survey at the same time points, including the same questions with exception of the questions relating specifically to the experience of the different steps of the PIE-approach.

As our primary outcome, the 17 items Positive Health measurement scale will be used, which is a 17-item measurement model based on the 44-items MPH dialogue tool. It has been developed to measure the effects of interventions on a broad perspective of health dimensions and uses a six factor structure, comprising the factors physical fitness, mental functions, future perspectives, contentment, social relations, and daily life-management (slightly different from the dimensions used in the dialogue tool) to provide a broad understanding of self-assessed health-related quality of life. An average score (1–10) for each factor is calculated. Higher scores are associated with better quality of life. The 17-items Positive Health measurement scale is in Dutch, developed by Dutch researchers and validated for use in the Netherlands [49] and derived from the concept of Positive Health. Positive Health is a concept of health proposed by Huber that describes health ‘as the ability to adapt and self-manage, in the face of physical, mental, and social challenges’ [40]. This allows to take a broader look at one’s perceived health than just as a ‘state of complete physical, mental and social well-being’ according to the World Health Organization [50]. In addition to this, the 1-item self-assessed general health (5 point Likert scale; very well-well-not well, not poor-poor-very poor) is used as an overall widely used indicator of health status [51].

Secondary outcomes will include questions on the experience of stress (adverse life events, financial stress (Psychological Inventory of Financial Scarcity [52], stress at home, work, or otherwise, and the handling of stress) and lifestyle behaviors (intake of fruit and vegetables, weekly exercise, hours of sleep, alcohol intake and smoking habits).

Questions on sociodemographic characteristics will include gender, age, level of education, income, employment status, country of birth for the participant and their parents, marital status, and household composition. Additional factors such as health literacy (Set of Brief Screening Questions [53] and digital skills (adapted version of the Digital Difficulties Scale [54]) will also be evaluated.

#### Qualitative data collection

Participants will be invited for a focus group discussion half a year after inclusion of a minimum of 20 participants, or when inclusion has ended (see Table 3). During the focus groups, the different program theories will be tested and refined according to realist interviewing principles [55, 56]. The main question concerns what generative causal mechanisms were triggered by the PIE-approach. The IPT for the four steps of the PIE-approach will be discussed in depth, how each step affected the participant and what contextual factors, facilitators, and barriers played a role in their experience and what mechanisms it triggered. The use of focus groups allows for the exploration of a plethora of different mechanisms and how participants view these in relation to others [57].

Semi structured in-depth interviews will be held with care professionals approximately 3 months after inclusion of 20 participants, or when inclusion has ended, to ensure that the professionals have enough time to gain valuable experience with the PIE-approach (see Table 3). In these realist interviews, the IPT will be tested and refined from the perspective of the care professional. In addition, reach, dose of the intervention delivered by care professionals, dose of the intervention received by participants, facilitators and barriers of the PIE-approach, and contextual factors that have influence on user engagement will be addressed.

For each pilot, on location field observations will be carried out on the content of care delivery, interactions between care professionals and participants, together with participants’ responses to the PIE-approach (see Table 3). Field notes together with policy and evaluation documents will inform the testing and refining of the IPT.

### Phase 4 Data analysis

### Quantitative analysis

For statistical analysis, the propensity score matching technique will be used as an alternative design to evaluate the effectiveness of the PIE-approach since a randomized controlled trial is not feasible or desired in this situation [58, 59]. Allocation of persons to the intervention and control group is not random, so differences between the intervention and control group may exist. The goal of the propensity score method is to balance two non-equivalent groups on observed covariates to get more accurate estimates of the effect of the PIE-approach. The propensity score is the probability of being exposed to the PIE-approach given the observed characteristics of a person [60, 61]. Propensity scores will be estimated for all individuals by regressing the participation status in the PIE-approach (yes/no) on the measured baseline characteristics using a logistic regression model.

Preferably, individuals participating in the PIE-approach and controls will be matched based on their propensity score (propensity score matching). Then, the intervention effects will be estimated comparing the primary, and secondary, outcomes between the participating and not-participating individuals in the matched sample. If the number of matched partners is less than anticipated, other propensity score methods (e.g. inverse probability of treatment weighting or regression adjustment) will be considered. We will use multiple regression models for the quantitative data using the matched data. Descriptive statistics will be used to describe the sociodemographic characteristics of the study sample and implementation results (such as reach, dose delivered and received).

Under the assumption of a power of 80% and a significance level of 5% (two sided), and a (norm-based) standard deviation of 1.79, we would be able to detect a difference of approximately 0.55 point in the score of one of the six domains of the 17 items MPH measuring scale with 175 participants with completed questionnaires and participating in the PIE approach. Due to possible mismatches between the propensity scores of the intervention and the control group, we aim for a sample of 200 respondents in both groups. Based on this calculation, we aim to recruit between 20 and 40 participants from each location for a maximum of 10 pilot locations.

#### Qualitative analysis

Qualitative data analysis will be an iterative process [62]. Data will be coded in Dutch to ensure credibility [63]. The realist qualitative data analysis and synthesis method of Gilmore et al. will be followed, including different phases of data analysis for identifying context-mechanisms-outcome configurations, subsequent synthesis and refinement of program theories [64]. Qualitative data analysis software NVivo will be used to assist coding qualitative data to allow for a transparent and traceable process [65, 66]. Extensive memos identifying the links between concepts and the emergence of CMO configurations will be written during the coding process [66].

### Phase 5: Refinement and validation of the program theory

The method of Gilmore et al. includes different phases, concluding with phases of synthesis and refinement of program theories according to the CMO configurations within pilot locations and across pilot locations [64]. This analysis of the different pilot locations will allow for comparison of CMO configurations generated in each location. In turn, this will lead to a broader understanding of the underlying causal mechanisms, and refinement and consolidation of the initial program theory. The refined program theory per pilot location will be discussed and validated with the respective local stakeholders. The final program theory will be presented in the online learning community to all stakeholders for validation.

## Ethics

Data collection for this study for the first pilot location was started in June 2021 and is expected to continue for the last pilot location until summer 2025. The Medical Ethical Committee of the Erasmus MC reviewed and approved this study protocol (MEC-2021-0364) and declared the Medical Research Involving Human Subjects Act not applicable to our study. Written or digital informed consent forms are obtained from participants (care professionals and participants) included for surveys, interviews and focus groups. To maintain confidentiality of the study participants, personal identifiable information is kept separated from study results and both are kept securely at the Erasmus MC. A unique respondent identification number is assigned to each study participant so that data can be processed anonymously. Study results will be written up and published in internationally peer-reviewed journals and will be presented at national or international conferences and to stakeholders in the learning community network.

## Discussion

The purpose of this article is to present a detailed description of the realist evaluation protocol of the PIE-approach for individuals with low SEP. The PIE-approach is a four-step approach aimed at promoting and assisting in behavior change to improve the participants’ health and health behaviors or address related barriers. The realist evaluation aims to understand what causal mechanisms are triggered within different contexts for participants that either help or impede them within this behavior change process.

Among the strengths of the PIE-approach and its evaluation are the realist evaluation design with mixed methods, multiple pilot locations and the focus on groups with a low SEP. The use of the realist evaluation method with the triangulation of quantitative and qualitative data from both the participants’ and professionals’ perspective ensures rich information on the different elements of the PIE-approach, its effectiveness and how this impacts participants. Not merely evaluating whether a complex intervention is successful but rather what causal generative mechanisms underlie its success or failure is needed to develop a broader understanding of how complex interventions work [67]. In addition, by the use of multiple locations in a real world setting, different implementation strategies, the experienced difficulties and impact can also be compared to gain a better understanding of mechanisms in different contexts. Our work may lead to more in depth understanding of blended care eHealth interventions for low SEP groups. This addresses a knowledge gap in this area highlighted by recent studies [31, 68].

The focus on participants with a low SEP is important, as it is very much needed to understand what contributes to diminishing health disparities between higher and lower SEP. However, we acknowledge that interventions solely aimed at the individual might stimulate the tendency to hold them fully accountable for their health status, even within extenuating circumstances beyond their control. The group with a low SEP faces adverse conditions such as financial hardship which will not be solved within this intervention [26]. It is vital that the environment and system also change to facilitate healthy behaviors and thus for the PIE-approach to optimally function. Unearthing the contextual factors that play an important role are a step in the direction to adequately address and further this systemic change.

There are also potential limitations to our study with regards to the use of practice based research, recruitment, and qualitative input. Conducting research in real world settings is subjected to societal challenges which can either stimulate or hamper such research. One factor such as the timing of the COVID-19 pandemic has impeded our research, as the pandemic hampered recruitment of pilot locations as priorities of policy makers and care professionals shifted due to more urgent challenges. Furthermore, sparse resources such as staff were directed elsewhere. The socio-political climate is equally important in setting up research for prevention. A recent increased focus on health disparities and prevention brings about more awareness and good will, which can facilitate implementation. Implementation of the PIE-approach is very much dependent on the willingness and resources of local social and care organizations.

Another limitation may lie in the qualitative response of participants. Some participants may feel apprehensive to criticize the intervention or to elaborate on their personal situations that inhibit health behavior change, resulting in more socially acceptable or desirable responses. This may introduce social desirability bias [69]. In order to limit this bias, the researchers are trained in the realist qualitative interviewing techniques with the use of rival theories and an open and inductive demeanor [55, 70]. The use of posing rival theories to the initial program theories allows for the exploration of intended and unintended consequences [70]. The researchers will emphasize an open atmosphere for sharing both positive and negative consequences during the interviews and focus groups and stimulate the group to share their different experiences.

## Conclusions

In this paper, we presented the protocol for the realist evaluation of the preventive integrated eHealth approach. The PIE-approach aims to assist in health behavior change among adults of low SEP through blended care by offering individually tailored insight in health with the use of an eHealth tool, personalized advice from a care professional, and accessible entry to local follow-up services. Following this protocol, we will execute a realist evaluation with mixed methods to evaluate different strategies of implementing the PIE-approach in real-world settings. This will produce a final program theory that elucidates the causal mechanisms across different contexts that act as barriers or facilitators from the perspective of participants and professionals. This will contribute to a broader understanding of the use of eHealth interventions for individuals with low SEP.

## Data Availability

No datasets were generated or analysed during the current study.

## Abbreviations

SEP: socioeconomic position
PIE approach: preventive integrated eHealth approach
CMO: context-mechanism-outcome
PHC: Personal Health Check
NIPED: Netherlands Institute for Prevention and EHealth Development
MPH: My Positive Health
IPH: Institution of Positive Health
IPT: initial program theory
GP: general practitioner
ISCED: International Standard Classification of Education
